# AgAnimalGenomes: browsers for viewing and manually annotating farm animal genomes

**DOI:** 10.1007/s00335-023-10008-1

**Published:** 2023-07-17

**Authors:** Deborah A. Triant, Amy T. Walsh, Gabrielle A. Hartley, Bruna Petry, Morgan R. Stegemiller, Benjamin M. Nelson, Makenna M. McKendrick, Emily P. Fuller, Noelle E. Cockett, James E. Koltes, Stephanie D. McKay, Jonathan A. Green, Brenda M. Murdoch, Darren E. Hagen, Christine G. Elsik

**Affiliations:** 1 Division of Animal Sciences, University of Missouri, Columbia, MO 65211 USA; 2 Department of Molecular and Cell Biology, University of Connecticut, Storrs, CT 06269 USA; 3 Department of Animal Science, Iowa State University, Ames, IA 50011 USA; 4 Department of Animal, Veterinary and Food Sciences, University of Idaho, Moscow, ID 83844 USA; 5 Department of Animal and Food Sciences, Oklahoma State University, Stillwater, OK 74078 USA; 6 Department of Animal, Dairy, and Veterinary Sciences, Utah State University, Logan, UT 84322 USA; 7 Department of Animal and Veterinary Sciences, University of Vermont, Burlington, VT 05405 USA; 8 Division of Plant Science & Technology, University of Missouri, Columbia, MO 65211 USA; 9 Institute for Data Science & Informatics, University of Missouri, Columbia, MO 65211 USA

## Abstract

Current genome sequencing technologies have made it possible to generate highly contiguous genome assemblies for non-model animal species. Despite advances in genome assembly methods, there is still room for improvement in the delineation of specific gene features in the genomes. Here we present genome visualization and annotation tools to support seven livestock species (bovine, chicken, goat, horse, pig, sheep, and water buffalo), available in a new resource called AgAnimalGenomes. In addition to supporting the manual refinement of gene models, these browsers provide visualization tracks for hundreds of RNAseq experiments, as well as data generated by the Functional Annotation of Animal Genomes (FAANG) Consortium. For species with predicted gene sets from both Ensembl and RefSeq, the browsers provide special tracks showing the thousands of protein-coding genes that disagree across the two gene sources, serving as a valuable resource to alert researchers to gene model issues that may affect data interpretation. We describe the data and search methods available in the new genome browsers and how to use the provided tools to edit and create new gene models.

**Supplementary Information:**

The online version contains supplementary material available at 10.1007/s00335-023-10008-1.

## Introduction

Genome resources for livestock species are being used to improve animal health and production efficiency, reduce environmental impact, mitigate disease, and increase fertility through technologies, such as genomic selection and genome editing (Georges et al. 2019; Tait-Burkard et al. 2018). Genomic selection accuracy can be improved using functional information to reduce the search space for causal variants, which are found in both coding and noncoding sequences (Georges et al. 2019; Giuffra et al. 2019). Thus, the aim of the Functional Annotation of Animal Genomes (FAANG) Consortium is to produce catalogues of functional elements for livestock species (Andersson et al. 2015). High-quality gene lists are essential for interpreting the functional annotation data, targeting genes of interest and elucidating biological mechanisms. Genes in assembled genomes are annotated using computational methods that leverage sources of biological evidence, such as transcriptome sequencing data. While the reference genome assemblies and transcriptome resources of several farm animal species have been considerably upgraded (Bickhart et al. 2017; Davenport et al. 2022; Kalbfleisch et al. 2018; Rosen et al. 2020; Warr et al. 2020; Warren et al. 2017), challenges in automated gene prediction can still lead to missing genes or erroneous gene models.

We have previously described genome annotation tools available at the Bovine Genome Database (BGD) that allow users to verify and manually refine genes of interest (Triant et al. 2020). We have expanded upon those tools and have created a new resource called AgAnimalGenomes that includes additional animal species of interest to the FAANG Consortium. The new tools, available at http://AgAnimalGenomes.org, are based on JBrowse (Buels et al. 2016) and Apollo (Dunn et al. 2019) and support bovine (*Bos taurus* ARS-UCD1.2), chicken (*Gallus gallus* GRCg6a), goat (*Capra hircus* ARS1), horse (*Equus caballus* EquCab3.0), sheep (*Ovis aries* ARS-UI_Ramb_v2.0), pig (*Sus scrofa* Sscrofa11.1) and water buffalo (*Bubalus bubalis* NDDB_SH_1). Advantages of the AgAnimalGenomes browsers compared to the existing Ensembl (Howe et al. 2021) and UCSC Genome (Navarro Gonzalez et al. 2021) browsers are the availability of hundreds of tissue-specific RNAseq tracks, each with a variety of visualization capabilities, and the capacity to edit and annotate novel genes and transcripts. These tools enable researchers to resolve disagreements between Ensembl and RefSeq gene models, correct exons that are incongruent with transcriptome evidence, extend partial coding or untranslated regions (UTR), and create novel gene and transcript isoform models. Furthermore, the browsers provide tracks representing tissue-specific functional sequence annotation data (ChIP-seq, ATAC-seq, DNase Hypersensitivity, and chromatin states) generated by the FAANG Consortium.

## Visualizing genomes and track data with JBrowse

All browser track data have been previously published (Tables 1 and 2) and are freely accessible for viewing using AgAnimalGenomes JBrowse without logging in. However, logging in to Apollo (described below) is required to access gene editing functions and view user submissions, as well as exporting any annotations. Apollo registration is free and is available using the *Apollo Registration* pulldown menu in the navigation bar. In this paper, we will first describe tools available without logging in and will then describe Apollo annotation tools.

**Table 1:** Genome, gene, and variation data sources

Data type	Species	Genome or data source	Reference
Genome	Bovine	ARS-UCD1.2	Rosen et al. (2020)
Genome	Chicken	GRCg6a	Warren et al. (2017)
Genome	Goat	ARS1	Bickhart et al. (2017)
Genome	Horse	EquCab3.0	Kalbfleisch et al. (2018)
Genome	Pig	Sscrofa11.1	Warr et al. (2020)
Genome	Sheep	ARS-UI_Ramb_v2.0	Davenport et al. (2022)
Genome	Water buffalo	NDDB_SH_1	Not published
Genes	Bovine, chicken, goat, horse, pig	Ensembl Genes	Cunningham et al. (2022)
Genes	All species	RefSeq Genes	O'Leary et al. (2016)
SNP	Bovine, chicken, goat, horse, pig	Ensembl Variation	Cunningham et al. (2022)
SNP	Sheep	European Variation Archive	Cezard et al. (2022)
QTL	Bovine, chicken, goat, horse, pig, sheep	AnimalQTLdb	Hu et al. (2022)

**Table 2:** Tissue-specific datasets

Data type	Species	Bioproject	Reference
RNAseq	Bovine	PRJEB14330	Kern et al. (2018)
		PRJEB25677	Chamberlain et al. (2015)
		PRJEB27455	Foissac et al. (2019)
		PRJEB35127	Dorji et al. (2020)
		PRJNA263600	Triant et al. (2020)
		PRJNA294306	Triant et al. (2020)
		PRJNA379574	Triant et al. (2020)
		PRJNA665193	Kern et al. (2021)
	Chicken	PRJEB12891	Kuo et al. (2017)
		PRJEB14330	Kern et al. (2018)
		PRJEB27455	Foissac et al. (2019)
		PRJNA204941	McCarthy et al. (2019)
		PRJNA665193	Kern et al. (2021)
	Goat	PRJEB23196	Bush et al. (2019), Muriuki et al. (2019)
		PRJEB27455	Foissac et al. (2019)
	Horse	PRJEB26787	Gao et al. (2020)
		PRJEB35307	Kingsley et al. (2019)
	Pig	PRJEB14330	Kern et al. (2018)
		PRJEB19268	Derks et al. (2018)
		PRJEB19386	Warr et al. (2020)
		PRJEB27455	Foissac et al. (2019)
		PRJEB37735	Pan et al. (2021)
		PRJNA311523	Li et al. (2017)
		PRJNA665193	Kern et al. (2021)
	Sheep	PRJEB19199	Clark et al. (2017)
		PRJEB6169	Clark et al. (2017)
		PRJNA414087	Davenport et al. (2022)
	Water buffalo	PRJEB25226	Young et al. (2019)
		PRJEB4351	Low et al. (2019)
Chromatin accessibility (ATAC-seq and DNaseI hypersensitivity peaks)	Bovine	PRJNA665194	Kern et al. (2021)
	Chicken	PRJNA665194	Kern et al. (2021)
		PRJNA665196	Kern et al. (2021)
	Goat	PRJEB27111	Foissac et al. (2019)
	Pig	PRJEB27111	Foissac et al. (2019)
		PRJNA665194	Kern et al. (2021)
Chromatin state	Bovine	PRJNA665212	Kern et al. (2021)
	Chicken	PRJNA665212	Kern et al. (2021)
	Pig	PRJNA665212	Kern et al. (2021), Pan et al. (2021)
CTCF binding (ChIP-seq peaks)	Bovine	PRJNA665197	Kern et al. (2021)
	Chicken	PRJNA665197	Kern et al. (2021)
	Pig	PRJNA665197	Kern et al. (2021)
Histone modification (ChIP-seq peaks)	Bovine	PRJNA665199	Kern et al. (2021)
		PRJNA665209	Kern et al. (2021)
		PRJNA665214	Kern et al. (2021)
		PRJNA665216	Kern et al. (2021)
	Chicken	PRJNA665199	Kern et al. (2021)
		PRJNA665209	Kern et al. (2021)
		PRJNA665214	Kern et al. (2021)
		PRJNA665216	Kern et al. (2021)
	Horse	PRJEB35307	Kingsley et al. (2019)
		PRJEB42315	Kingsley et al. (2021)
	Pig	PRJEB37735	Pan et al. (2021)
		PRJNA665199	Kern et al. (2021)
		PRJNA665209	Kern et al. (2021)
		PRJNA665214	Kern et al. (2021)
		PRJNA665216	Kern et al. (2021)

## Genome navigation

**Fig. 1:**
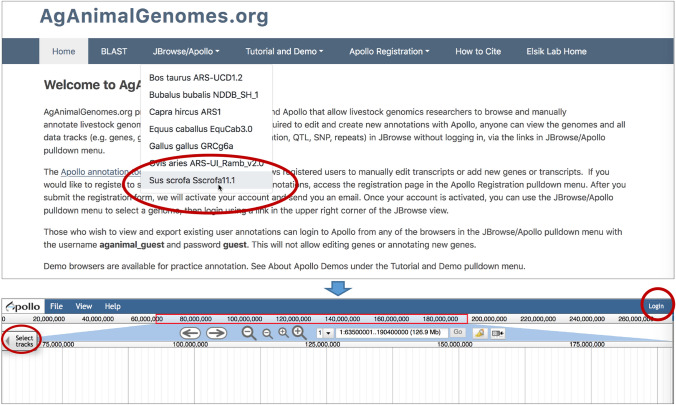
Accessing JBrowse using the *JBrowse/Apollo* pulldown menu. When you select a genome in the pulldown menu, JBrowse opens, but may not display any tracks. The top number line shows the coordinates along the entire chromosome, with the range of the current view shown in the red rectangle. The lower number line displays the coordinates of the current view. Arrows for panning left and right and buttons for zooming in and out are above the browser, to the left of the pulldown menu that lists the chromosomes in descending order by length. The search box can take coordinates, gene symbols, and identifiers. Tracks for visualization are selected using the *Select tracks* button, circled in red on figure left. The login button circled on figure right provides access to the Apollo annotation tool

Genome browsers are accessed using the *JBrowse/Apollo* pulldown menu in the navigation bar at the top of the home page (Fig. 1). Once the JBrowse page for an organism is loaded for the first time, you will see a blank viewer with number lines and navigation controls at the top of the page (Fig. 1). The upper number line represents coordinates of an entire chromosome and has a red rectangle showing the specific location of the current view. The lower number line represents the chromosomal coordinates of the current view. Arrow buttons allow panning left and right, and zoom levels can be adjusted using the plus and minus icons. To the right of the zoom buttons is a pulldown menu to select a chromosome or scaffold, which are provided in order of descending length, and a box to enter chromosome coordinates. Rather than selecting a chromosome and entering coordinates, you can enter a gene symbol, Ensembl or RefSeq transcript or gene identifier, or QTL trait name to navigate directly to its genome location. When the entered text is found in more than one chromosomal region, a table appears that allows you to select one of the locations.

## Faceted track selector

**Fig. 2:**
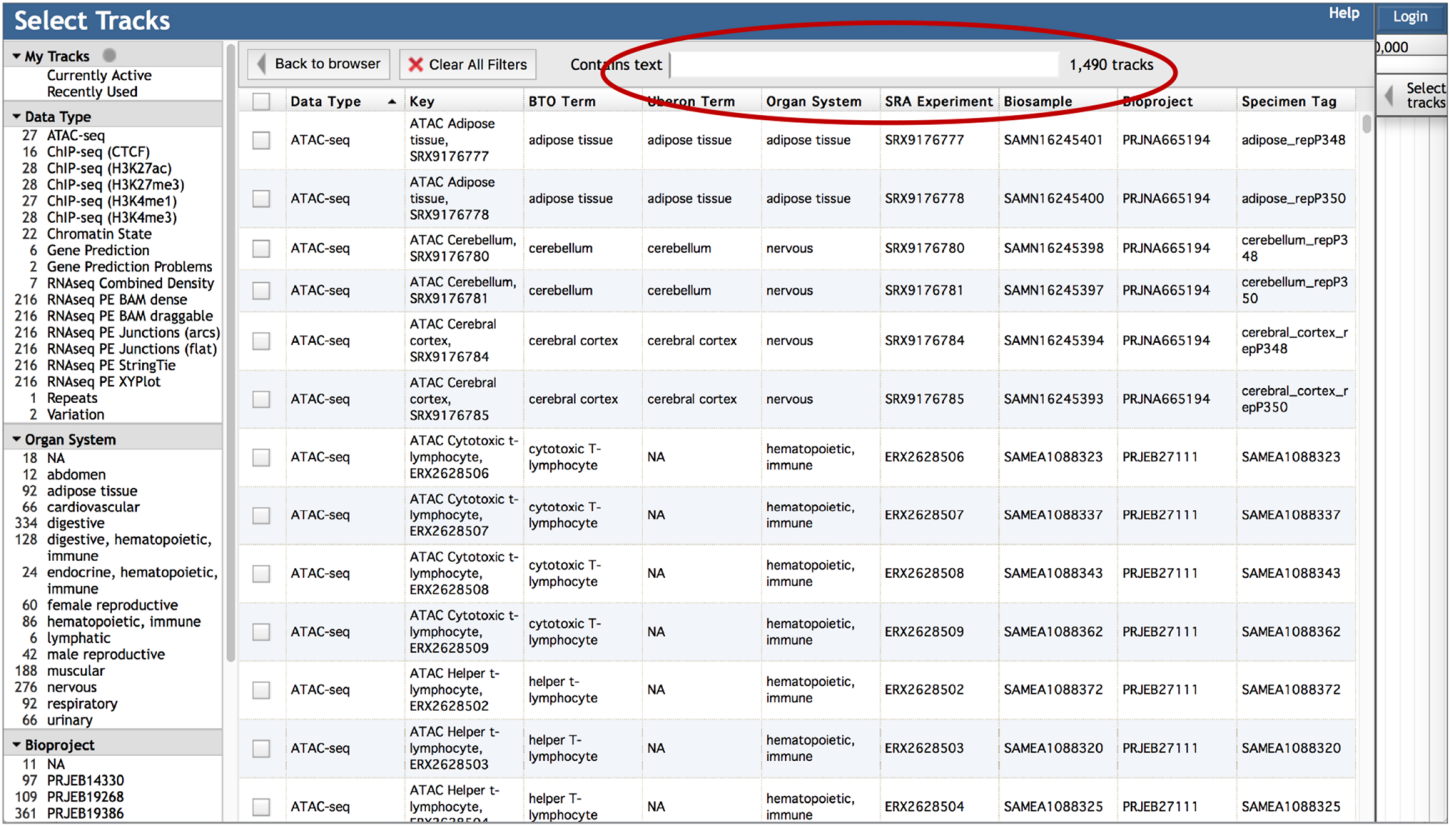
The Faceted Track Selector for the *S. scrofa* browser. The panel on the left provides the tracks organized by Data Type, Organ System, and Bioproject. Selecting one or more categories filters the table on the right. The table can be further filtered by entering text in the box shown circled in red on the figure. Tracks are selected by clicking the small boxes on the left of the table

To select tracks for viewing, click the *Select tracks* button in the upper left of a JBrowse window (Fig. 1). This brings up the Faceted Track Selector where tracks are organized into categories, based on Data Type and, for tissue-specific tracks, Organ System and Bioproject (Fig. 2). Highlighting one or more categories on the left panel filters rows in the table on the right, which provides metadata, including tissue, SRA experiment accession, Biosample accession, Bioproject, Brenda Tissue Ontology, and Uberon Ontology terms. For species with data from multiple experiment types (e.g., RNAseq, ChIP-seq, ATAC-seq), an additional attribute called *Specimen Tag* allows you to identify all tracks for an individual sample. The table is further searchable by entering any metadata text in the *Contains text* search box above the table. Tracks are selected for viewing by clicking boxes to the left of the table.

## Browser tracks

### Gene predictions

**Fig. 3:**
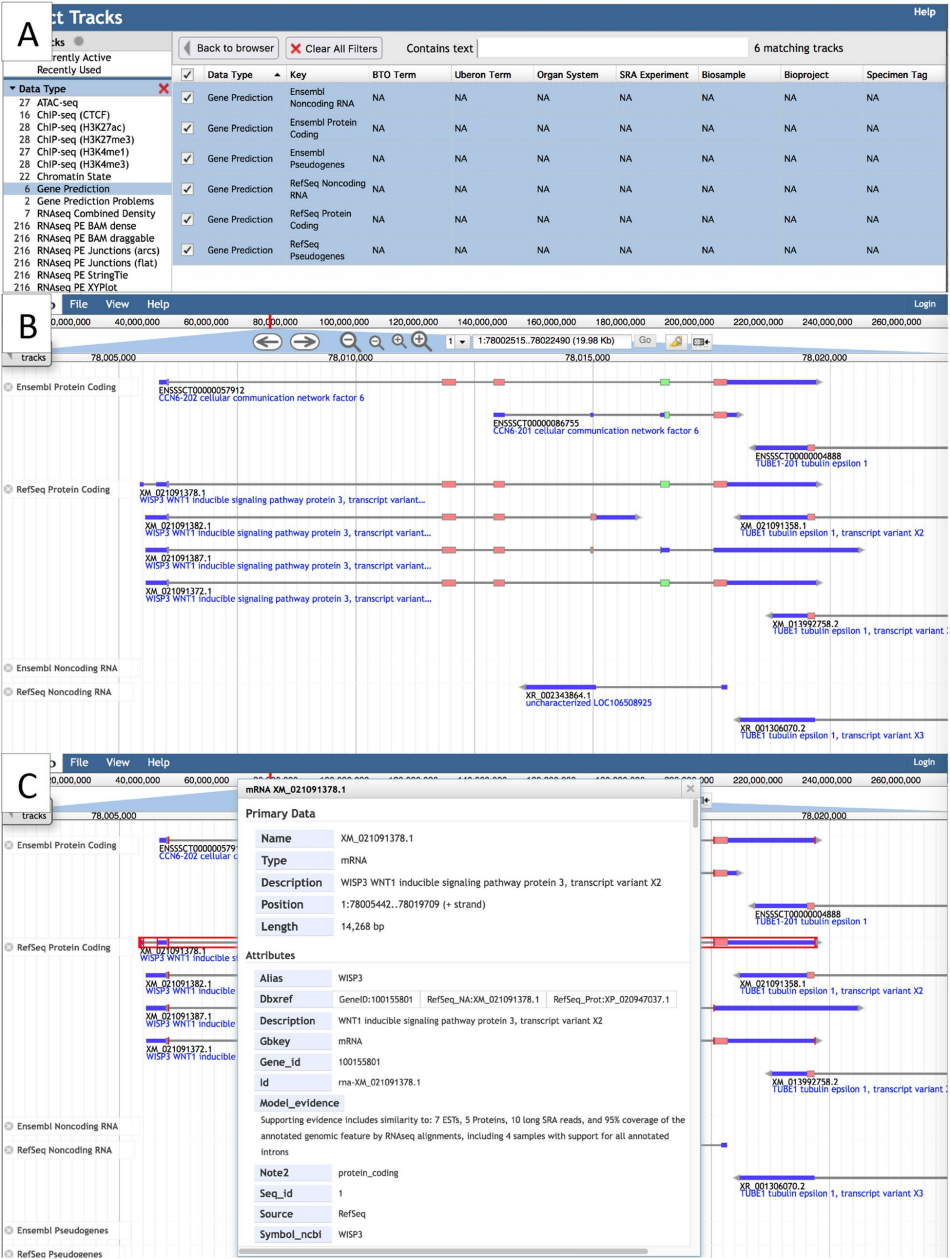
**A** The Faceted Track Selector after selecting the Gene Prediction category on the left to filter for gene prediction tracks. **B** *Sus scrofa* JBrowse view after selecting Ensembl and RefSeq gene tracks. Exons are color coded, with non-coding exons (UTR and noncoding transcripts) in dark blue and CDS in three possible colors, depending on the reading frame (only two colors are shown here). **C** Right clicking a transcript allows you view details

Gene prediction tracks are provided for RefSeq and Ensembl genes (Fig. 3). They display transcripts and are divided into protein-coding genes, noncoding RNA genes, and pseudogenes. In our browsers, the gene prediction tracks are color-coded and configured to show untranslated regions (UTR) and noncoding transcripts in dark blue. The coding portions of the exons are slightly thicker than the UTR and appear in different colors representing reading frames. An arrow at one end of the transcript indicates coding direction, pointing right for coding on the plus strand, and left for coding on the minus strand. When zoomed out, gene prediction tracks appear as histograms depicting gene density, while zooming in allows the visualization of predicted introns and exons. Right clicking a transcript feature provides more information, which may include gene symbol, description, and database cross-references.

### Problematic genes

**Fig. 4:**
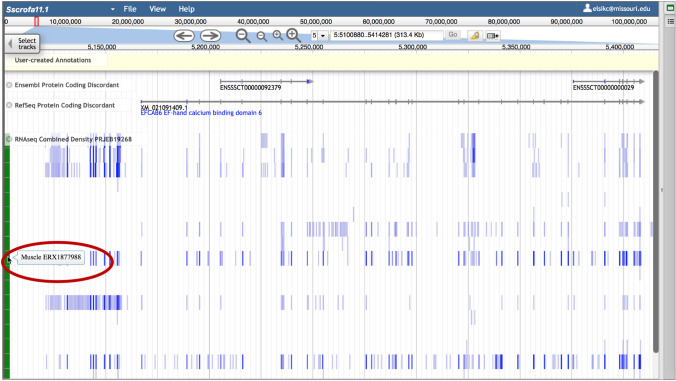
* Sus scrofa* JBrowse view showing a split/merge example in the *Ensembl* and *RefSeq Protein-Coding Discordant* tracks. This locus has one RefSeq gene but two different Ensembl genes. Below the gene prediction tracks is a *RNAseq Combined Density* track, with the cursor hovering over the label (circled in red on the figure) of an experiment that shows signals at the exons of interest, particularly those in the RefSeq gene that are not present in Ensembl. The experiment near the bottom of the combined track also provides information at the discordant exons

To highlight problematic genes as candidates for manual annotation, we have created tracks for Ensembl and RefSeq genes that disagree with each other (only for species with both gene sets) (Table 3). Discordant gene loci include genes that appear in one gene set but not the other or have split/merge differences, i.e., when genes in one gene set appear to be split or merged compared to genes at the same location in the other gene set (Fig. 4). These tracks are available under the *Gene Prediction Problems* category in the Faceted Track Selector.

**Table 3:** Numbers of discordant protein-coding genes

Organism	Ensembl	RefSeq
Bovine	3122	2466
Chicken	2163	2670
Goat	3264	2671
Horse	2716	3617
Pig	3371	2119

### RNA expression

RNAseq tracks are formatted in seven different track types that provide different visualizations depending upon your objectives. The *Combined Density RNAseq* tracks (Fig. 4) is used to identify RNAseq experiments that are informative for the gene of interest. A single Combined Density track provides heatmaps representing gene expression levels in log scale for all RNAseq experiments of a particular bioproject. Mousing over the legend on the left side of the browser, appearing as a green vertical bar, shows the tissue and accession of an individual experiment. Some bioprojects include hundreds of RNAseq experiments and their Combined Density tracks may take several moments to appear on the browser once selected.

**Fig. 5:**
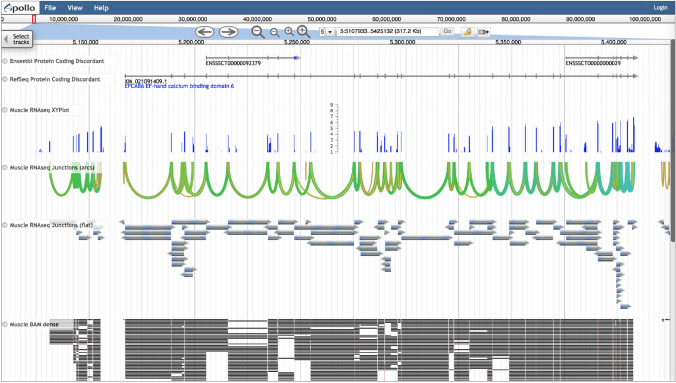
* Sus scrofa* JBrowse view showing different visualizations of the same RNAseq data

XYPlot tracks show read depth in log scale and are used to identify regions of high or low expression (Fig. 5). RNAseq junction tracks are available as arcs or as flat tracks (Fig. 5) and are useful for checking whether two exons should be connected in the same transcript and to confirm splice junctions. The arc version collapses junctions from individual reads for viewing when zoomed out to see entire genes or regions between genes. Arc thickness is related to the number of reads supporting the splice junction. The flat version collapses junctions from individual reads into a single bar and provides the number of reads that support the junction as the *Score* when viewing details about the feature.

BAM tracks showing read alignments are available in two visualizations which we call *dense* and *draggable*. The dense BAM track enables viewing a larger region than the draggable track (Fig. 5). Right clicking an individual read alignment in either a dense or draggable BAM track reveals additional information, such as the read sequence and quality, alignment score, and details about matches, mismatches, and indels in CIGAR format. The draggable BAM track is very computationally intensive and requires a sufficient zoomed-in level to avoid an error message (Fig. S7). The name, draggable, applies to an Apollo feature, described below.

StringTie tracks show transcript models created by assembling RNAseq read alignments. They can reveal the possibility of new isoforms (Fig. S2A). The tracks are similar in appearance to gene prediction tracks, but they do not distinguish UTR from coding regions. Similar to gene prediction tracks, StringTie tracks appear as histograms depicting density when zoomed out and as intron/exon structures when zoomed in (Fig. S2B).

### Functional sequence annotation

**Fig. 6:**
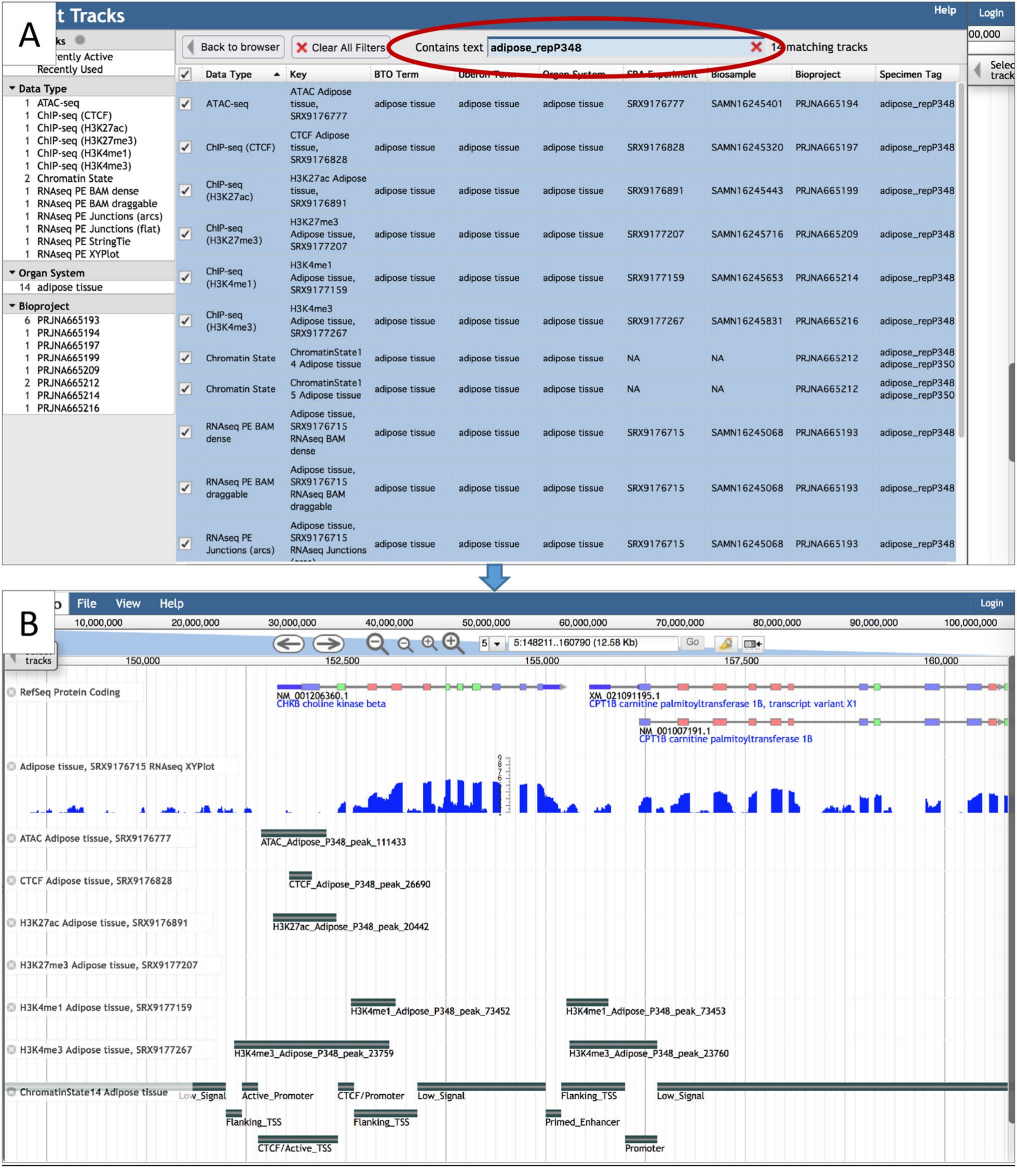
**A** *Sus scrofa* Faceted Track Selector showing the selection of all the tracks for an individual tissue sample by searching with the Specimen Tag (circled in red on the figure). **B** *Sus scrofa* JBrowse view showing FAANG data tracks for RNAseq, peaks for ATAC-seq, ChIP-seq peaks (histone marks and CTCF-binding sites), and predicted chromatin states

ChIP-seq data in AgAnimalGenomes are available for bovine, chicken, goat, horse, and pig; ATAC-seq data are available for bovine, chicken, goat, and pig (Table 2). Tissue-specific ATAC-seq peaks and ChIP-seq peaks for histone modification marks (H3K27ac, H3K27me3, H3K4me1, H3K4me3) and CTCF-binding sites appear as thick bars (Fig. 6). Right clicking an individual bar shows additional information, including the *Score*, which is the number of reads in that peak. To view tracks of all functional sequence annotation and RNAseq experiments for an individual sample, you can filter tracks in the Faceted Track Selector based on the *Specimen Tag* found in the far-right column for the species with sequence annotation experiments (all species except sheep and water buffalo) (Fig. 6).

### Chromatin states

Chromatin states for bovine, chicken, and pig are from published datasets (Kern et al. 2021; Pan et al. 2021) (Table 2). Chromosomal regions with particular tissue-specific states are shown as thick bars which are labeled with the state according to the specific terminology used in each publication (Fig. 6). The pig browser includes tracks for both 14-state (Kern et al. 2021) and 15-state datasets (Pan et al. 2021), while the bovine and chicken browsers each include a track for a 14-state dataset (Kern et al. 2021).

### Variation

Tracks in the Variation category representing quantitative trait loci (QTL) from AnimalQTLdb and sequence variants from Ensembl Variation or the European Variation Archive allow users to view this information in the context of genes and tissue-specific expression levels. Right clicking a variant id reveals the alternate and reference alleles and, for some species, the location of the variant relative to Ensembl genes. QTL features are labeled with traits. Right clicking a QTL feature reveals additional information, such as the breed, flanking markers, peak centimorgans, test statistics, model tested, test base, and PubMed ids. The QTL id provided in the information panel allows you to look for more information at AnimalQTLdb (https://www.animalgenome.org/cgi-bin/QTLdb/index) (Hu et al. 2022).

### Repeats

Repeats identified with RepeatMasker (Smit 2013–2015) can provide clues about potential assembly and gene prediction issues. Repeat features are labeled with a name, and right clicking the feature shows the repeat class and family.

### Private user tracks

You can view your own tracks using *Open Track File or URL* in the FILE pulldown menu. Your file is not uploaded to AgAnimalGenomes.org, but is viewed only in your local instance.

## Using BLAST with the browsers

AgAnimalGenomes has two sequence comparison tools that can be used to search the genomes. BLAT (Kent 2002) is built into Apollo (described below), while BLAST (Altschul et al. 1990) is external to JBrowse and Apollo, and results can be viewed in either JBrowse or Apollo. The BLAST tab in the AgAnimalGenomes main navigation bar leads to a BLAST menu based on SequenceServer (Priyam et al. 2019). You can conduct BLAST searches against the genome assemblies using BLASTN (for nucleotide queries) or TBLASTN (for protein queries) (Fig. S3A). The *Advanced parameters* box allows you to modify BLAST parameters, such as the *e*-value threshold, which has a default setting of 1*e*−5. You should increase the *e*-value for very short sequences, such as microRNA, or you may want to decrease the *e*-value if the results include too many paralogs. The results page provides a graphical overview of all hits, followed by a list of hits and then graphical views and alignments for each chromosome in the hit list. Clicking *View in JBrowse* (Fig. S3B) above the alignments allows you to view the BLAST High Scoring Pairs (HSPs) in JBrowse or Apollo (if you are already logged into Apollo, as described below) (Fig. S3C). In Apollo, you can drag individual BLAST HSPs to the Editing Area to start a new annotation (described below).

## Apollo annotation interface

In order to log in to Apollo, you must register for an account by selecting an organism from the *Apollo Registration* pulldown menu in the AgAnimalGenomes main navigation bar. The *Click here to register* link provides a menu prompting entry of your full name and email address (which serves as username) and desired password. Once the form is submitted, the AgAnimalGenomes administrator grants the user read, write and export access after verifying the email address, and notifies the user that the account has been activated. Apollo uses the email addresses as the owner labels for specific gene models, allowing communication between users who share interests in the same genes. A guest login that allows read and export functions is available by selecting *About Apollo Registration* in the *Apollo Registration* pulldown menu.

**Fig. 7:**
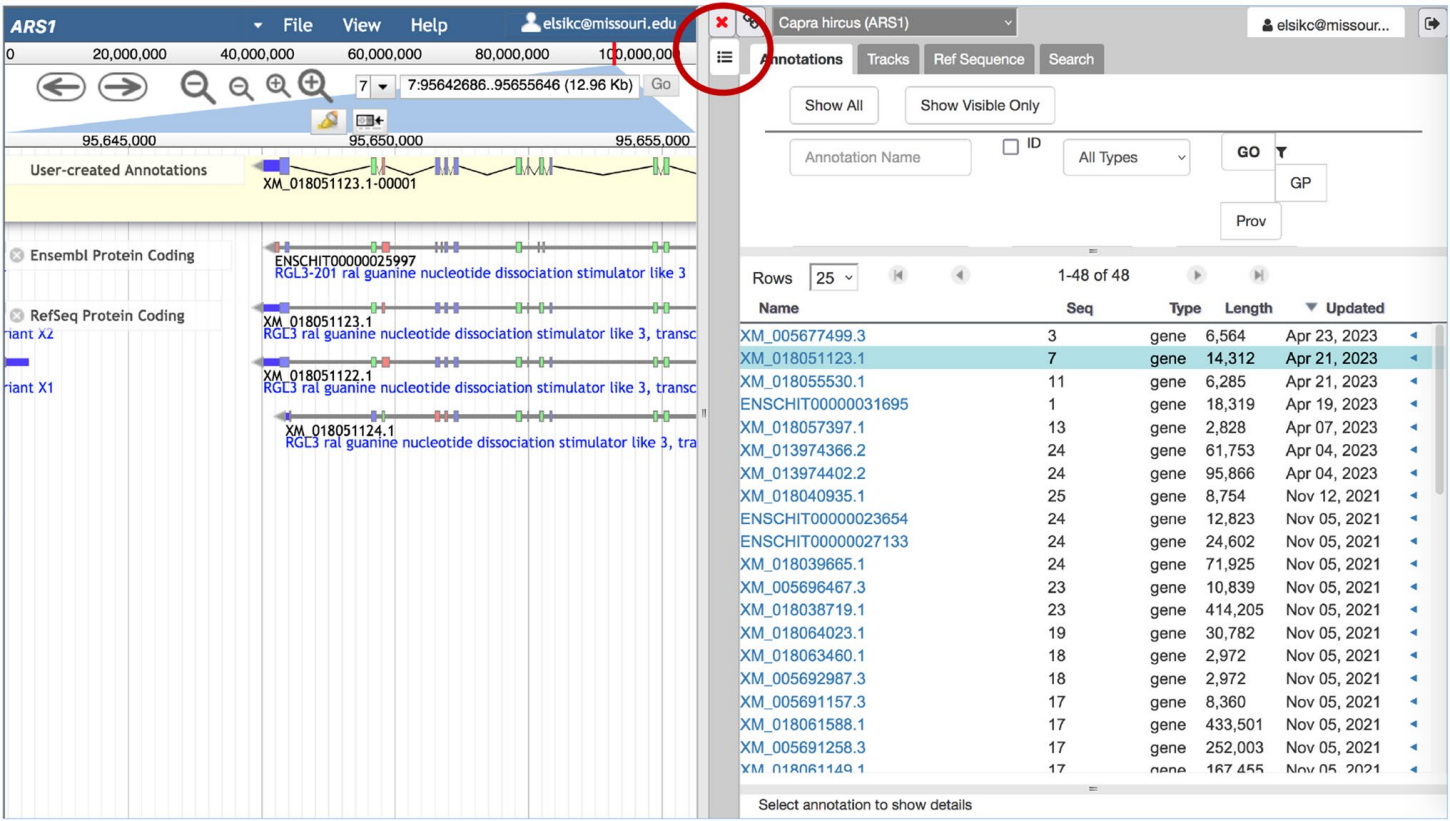
Apollo interface. On the right is the Information Panel with tabs for *Annotations*, *Tracks*, *Ref Sequence* (chromosome ids), and BLAT *Search*. The Information Panel can be hidden by clicking the red X in the upper left of the panel (circled in red on the figure). When the Information Panel is closed, the red X is replaced by a green-bordered square icon that can be used to re-open the Information Panel. The JBrowse *Select tracks* button that provides access to the Faceted Track Selector can be toggled on by clicking the list icon right below the red X (circled in red on the figure)

After you have been notified of your account activation, you can access genome annotation editing with Apollo by selecting a genome in the *JBrowse/Apollo* pulldown menu and then logging in to Apollo using the button in the upper right corner of the browser window. After logging in, the window appears split between the genome browser on the left and the *Information Panel* on the right (Fig. 7). The four tabs in the Information Panel include (1) the *Annotations* tab, which provides a list of submitted annotations and allows navigation directly to the location of a selected annotation (Fig. 7); (2) the *Tracks* tab, which allows track selection, but is not organized like the Faceted Track Selector; (3) the *Ref Sequence* tab which lists all chromosomes and unplaced contigs; and (4) the *Search* tab which allows you to perform a BLAT search to identify a genomic region based on a nucleotide or protein sequence. To hide the Information Panel and expose more of the genome browser, click the red X at the upper left of the panel (Fig. 7). To bring back the Information Panel, click the green-bordered square icon (which replaces the red X when the panel is closed), in the upper right corner of the browser. The genome browser in Apollo is split vertically between the upper *Editing Area* and the lower *Evidence Area*, where tracks will appear. To access the JBrowse Faceted Track Selector (described above), click the icon that resembles a list (Fig. 7), under the icon used to open or close the Information Panel, and the Select Tracks tab will appear at the upper left of the browser. Alternatively, you can click on the Tracks tab in the Information Panel and then check the box next to *JBrowse Selector*.

**Fig. 8:**
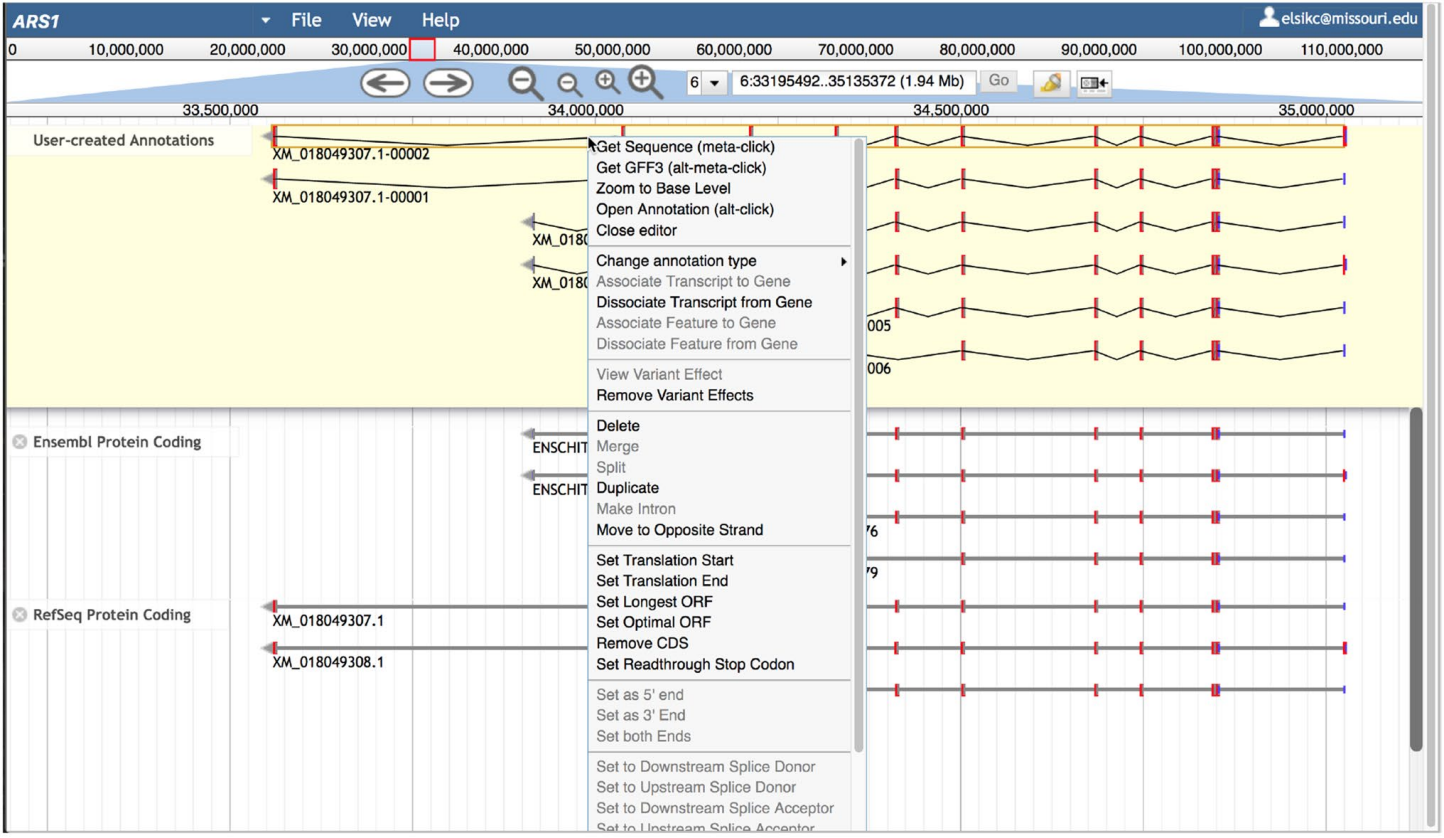
Apollo view showing annotations in the Editing Area. The top transcript has been right clicked to show a pop-up menu with editing options. Clicking the transcript also highlights the exon edges of the selected transcript and all matching exon edges of other transcripts in red. The *Undo*, *Redo,* and *Show History* options are available at the bottom of the menu and are not shown here

A gene annotation is initiated by dragging evidence from the Evidence Area to the Editing Area. Draggable evidence includes transcripts (RefSeq, Ensembl, and StringTie), aligned RNAseq reads from draggable BAM tracks, and BLAST HSPs. Once added to the Editing Area, the feature is considered an *annotation* or *gene model* and is automatically assigned a name and an owner (the user who initiated the annotation). The gene name and names of all transcripts annotated within the gene are based on the identifier of the first transcript added to the Editing Area for that gene, with digits added to create a unique name for each transcript variant (Fig. S4). The annotation can be altered in various ways, including modifying translation start and splice sites, adding exons, and adding or extending UTR. An exon boundary can be modified by dragging the boundary to the left or right. Right clicking the annotation provides a pop-up menu with various editing options, including *Merge*, *Split*, *Make Intron*, *Set Translation Start*, *Set Translation Stop,* and *Set Read-through Stop Codon* (Fig. 8). Selecting *Get Sequence* allows you to obtain the protein, coding, or cDNA sequence, and *Get GFF3* allows you to obtain the annotation in GFF3 format. You can see a list of modifications made to the annotation using *Show History* and can undo or redo previous changes. *Open Annotation* opens the Annotation tab in the Information Panel on the right, which allows you to add information about the gene or transcript (described below). The *Delete* option is functional for the annotation owner and the Apollo administrator but users are not able to delete annotations created by other users.

Visual cues to help with exon editing include corresponding red highlights at exon boundaries in other tracks when they agree with the exon boundaries of the selected annotation (Fig. 8) and a change in the color of an exon when a modification changes the reading frame. Modifications that introduce early stop codons cause one or more coding exons to change in appearance to be represented as UTR, depicted as thinner dark blue rectangles. Non-canonical splice sites are indicated by exclamation marks (Fig. S5).

User annotations and their modification histories are saved in real time on the server and are immediately viewable by others logged in to the Apollo instance. Furthermore, they are added to the Annotations tab of the Information panel, making them easy to find in future sessions. Within the Annotations tab is a table of annotations that can be searched by name, filtered by gene type and information, such as Gene Ontology if available, and sorted by chromosome, length, and date (Fig. 7). By default, the annotation table shows names of the gene annotations. Clicking a small arrow on any row to the very right of the annotation table shows the transcript names for a single gene. You can navigate to an annotation in the browser using the arrow immediately to the right of the transcript name or by clicking *Go* in the *Details* panel, which is opened by clicking a gene or transcript name (Fig. 7). Information in the Details panel includes the chromosomal location, owner, and dates created and last updated. The annotation name can be edited and then clicking *Sync name with transcript* will change the name on the browser. Gene symbol and description can also be added or edited. The *ID* button shows a permanent unique database identifier and provides a link for sharing the annotation with other users. Additional tabs in the Annotation Panel allow you to add Gene Ontology terms, gene products, database cross-references, comments, and other attributes. The *Provenance* tab allows you to add information indicating why other information was added or changed.

## Gene annotation process

We have previously described the general process of annotating a protein-coding gene, emphasizing the use of long-read transcriptome evidence to resolve split/merge discordances between Ensembl and RefSeq genes (Triant et al. 2020). Here, we focus on short read RNAseq, because long-read transcript data are not available for all species. We also update our approach for validating splice sites using RNAseq due to changes in the Apollo software. A detailed example and demonstration browsers for practice annotation are available on the AgAnimalGenomes website, under the *Tutorial and Demo* pulldown menu.

### Identify gene of interest

After logging in to Apollo, you must navigate to the subject gene locus. Annotators choose subject genes based on various criteria, such as research interest, presence in a *Gene Prediction Problems* track, or proximity to a QTL of interest. If you are interested in a specific gene, you may be able to locate it by entering a gene symbol, gene id, or transcript id in the browser search box. If the search for gene symbol or id yields no results, you can use either AgAnimalGenomes BLAST or the built-in Apollo BLAT tool to search the genome using a sequence retrieved from an external database, such as GenBank (Sayers et al. 2023b), Ensembl (Cunningham et al. 2022), or UniProt (UniProt Consortium 2023).

### Select gene tracks and add transcripts to editing area

Once you have navigated to the gene locus of interest, select gene evidence tracks for viewing using the Faceted Track Selector. Select both the RefSeq and Ensembl gene sets (if available). Agreement across the gene sets in transcript exon/intron structure provides support that the gene predictions are correct. In this case, drag transcripts from either RefSeq or Ensembl to the Editing Area. Sometimes the RefSeq and Ensembl genes disagree with each other due split/merge issues, in which case you should use RNAseq tracks, as described below, to decide whether to use a Refseq or Ensembl transcript to initiate the annotation.

As many isoforms as possible should be annotated for each gene. You can check for the possibility of additional isoforms using StringTie tracks after viewing the Combined RNAseq Density tracks to identify informative individual RNAseq tracks. Zooming into the gene will increase the intensity of the blue color in the Combined RNAseq Density track to reveal whether RNAseq reads exist in regions of interest. Identify the tissues or experiment ids for tracks with sufficient RNAseq evidence by mousing over the green vertical bars to the left of the track and then use this information to select individual StringTie tracks. Drag any new candidate isoform from the StringTie tracks to the Editing Area.

### Select and view RNAseq tracks

After candidate transcripts have been added to the Editing Area, use RNAseq tracks to decide whether to keep, delete, or edit the annotations. View one or more Combined RNAseq Density tracks, as described above, if you have not already done so. As you look for informative RNAseq tracks, focus on the exon regions, especially problematic regions, such as where exon/intron structure differs between Ensembl and RefSeq. The next step is to visualize the informative individual RNAseq tracks. Which type of RNAseq track you first view is a matter of preference and we recommend exploring all options to see what might be most suitable for your needs. The *RNAseq Junctions (arcs)* tracks are useful to determine the presence of introns while zoomed out to the entire gene. The *RNAseq BAM (dense)* tracks may require a more zoomed-in view to see whether RNAseq junctions correspond with an annotation intron, but have the advantage of showing read depth as a measure of support for the intron. The *RNAseq BAM (draggable)* tracks are similar to the dense BAM tracks, except the draggable tracks allow aligned reads to be dragged to the Editing Area to initiate or modify annotations and require further zooming in to avoid the *Too much data to show* error. It is helpful to remove the unspliced RNAseq reads from either the dense or draggable BAM tracks by mousing over the track label, clicking the arrow within the label, and then checking *Hide unspliced reads* near the bottom of the menu.

### Validate splice junctions

**Fig. 9:**
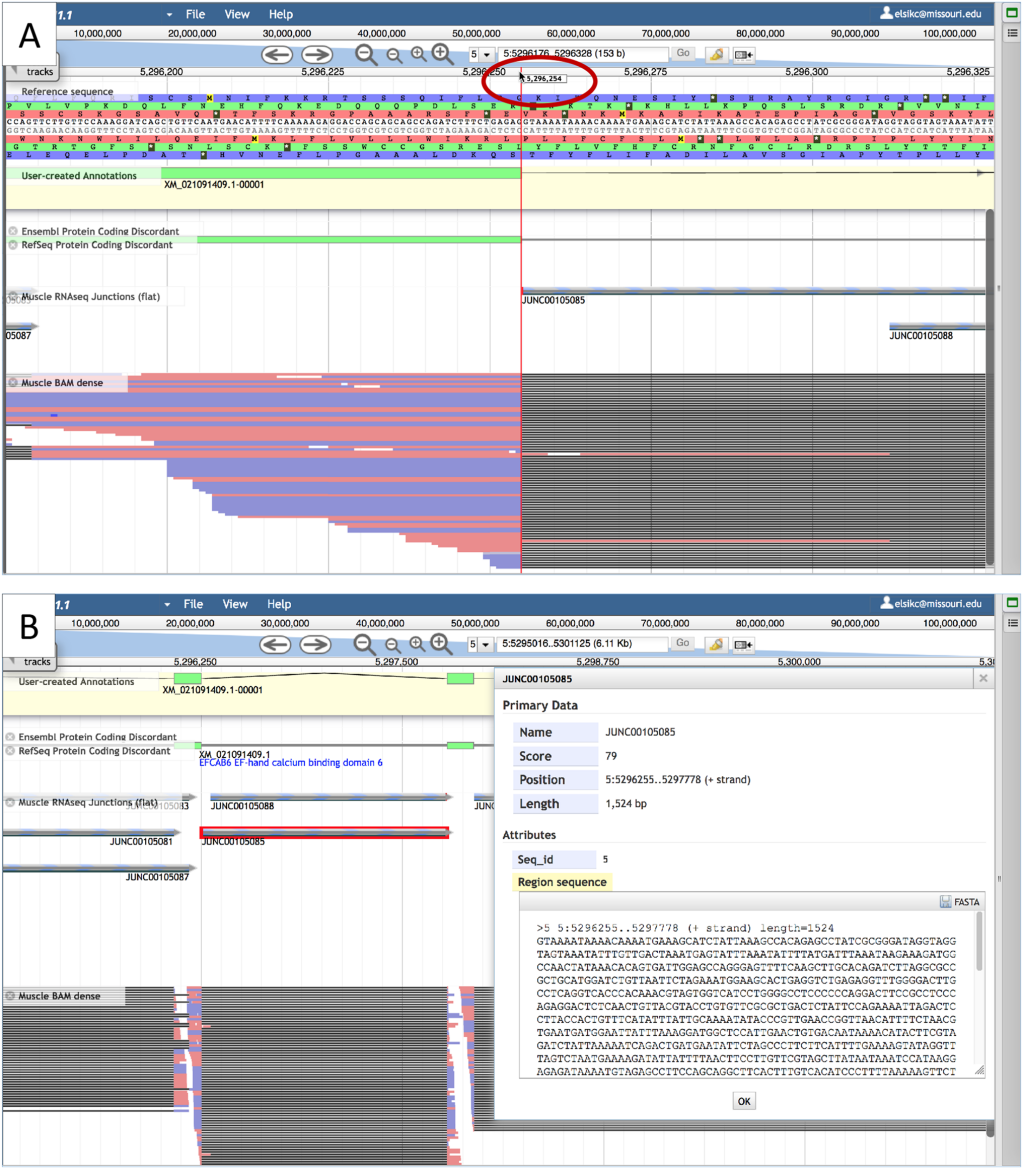
Validating a splice site. **A** A zoomed-in view with the cursor (circled in red) in the lower number line. The red line is lined up with the edge of the exon. The coordinate of the exon edge is shown in the rectangle next to the cursor (circled in red on the figure). **B** The information available when right-clicking the junction highlighted in red. Notice that the start coordinate of the junction is one higher than the coordinate of the exon edge shown in the upper panel, so this splice site is confirmed by 79 RNAseq reads (shown as the *Score* in the information panel)

After visualizing one or more RNAseq experiments in either arc junctions or dense BAM format, select one experiment to validate splice sites using flat RNAseq junctions (Fig. S5). The first step in splice site validation is to identify a single junction with edges that look like they correspond perfectly with an intron in the annotation. Zoom in to the exon boundary at one end of the intron until you can see the DNA sequence. When the cursor is positioned within the number line just above the DNA, a red line will appear along with a number showing the exact chromosome location (Fig. 9). Move the cursor until the red line perfectly overlaps the exon boundary and note the coordinate. Then right click the selected junction feature within the flat RNAseq Junctions track to view the coordinates and the number of reads supporting the junction. The coordinate corresponding with the splice site in question should be one larger or smaller than the coordinate of the exon edge, depending on whether you are viewing the splice donor or acceptor. After confirming the splice site at one end of the intron, move to the other end and use the same procedure with the same junction feature. The process of validating splice sites should be repeated for any exon/intron region that is discordant between RefSeq and Ensembl or any new intron within a candidate StringTie transcript. Each junction should be supported by multiple reads, the more the better.

### Edit annotations

Occasionally the annotations will need editing. Sometimes the coding region of the annotation changes when a RefSeq or Ensembl transcript is dragged to the Editing Area. By default, Apollo computes the coding region based on the largest open-reading frame, which may not be the same as the original coding sequence from RefSeq or Ensembl. If the translation start site in the annotation has changed, you can modify it by zooming in to the location of the original site, right clicking the first nucleotide of the start codon (the “A” of ATG), and selecting *Set Translation Start* in the menu. The translation stop codon can be reset in a similar manner.

Differences in exon colors between transcripts indicate differences in reading frames, which need to be corrected. Often resetting the translation start site solves the problem. If the translation start is correct, the reading frame difference is due to differences in exon boundaries. This sometimes occurs when BLAST HSPs are dragged to the Editing Area to annotate new exons. Figs. S6 through S8 show the process of dragging BLAST HSPs to annotate new exons (Fig. 6A), merging the exons to create a transcript (Fig. 6B and C), identifying exon boundary errors (Fig. S7) and making corrections (Fig. S8). You can quickly check the agreement of exon boundaries between transcripts by clicking the intron of a transcript. A red mark will appear at exon boundaries in other transcripts that agree with the clicked transcript. The zoom level should be sufficient to distinguish the red marks on each side of an exon (Fig. S7B). Check each exon starting at the first exon of the annotation. For any exon with boundaries that do not agree with others, zoom in to the DNA level and drag the exon boundary until it is concordant. After ensuring that the exon boundaries and start coordinate are correct, the exon colors should match between annotated transcripts and the genes in the Evidence Area (Fig. S8).

### Obtain sequence and perform BLAST search to known proteins

As previously described (Triant et al. 2020), once a protein-coding gene annotation is complete, each new or modified isoform should be compared to a well-curated protein sequence database to check for congruency with known proteins. The sequence of an annotation is obtained by right clicking it and selecting *Get Sequence*. The first choice of database to search is the well-curated UniProtKB/Swissprot database using BLAST at either the UniProt (https://www.uniprot.org/blast) or NCBI website (https://blast.ncbi.nlm.nih.gov/Blast.cgi) (Sayers et al. 2023a; UniProt Consortium 2023). If there is no match with a significant *e*-value (< 1*e*−05) in UniProtKB/Swissprot, the next database to try is the Model Organisms (landmark) database at NCBI. If that fails, select the RefSeq Proteins database and exclude your organism of interest from the search. Although RefSeq includes computationally predicted and hypothetical proteins, an alignment to a homologous protein from another organism provides support for the annotation. An alignment that covers the full length of both the annotated protein and the database protein sequence suggests the annotation is correct. An alignment that encompasses the full length of an annotated protein sequence but only part of a database protein suggests that the annotation is truncated. You may be able to correct the annotation with additional evidence, but if there is not sufficient evidence the issue can be noted in the Annotation Information Panel under the *Comment* tab. A partial alignment of an annotated protein to a database protein suggests the annotation has a reading frame shift or was extended incorrectly. Aligning the coding sequence (CDS) to the protein database will reveal whether the problem is due to a reading frame shift. Further annotation editing should be performed to correct the reading frame. If an incorrect extension was due to the merging of two genes, you should edit or redo the annotation. Any unresolved issues should be entered in the Comment section of the Annotation Information Panel.

Sometimes you will find that your selected gene of interest is perfectly congruent between RefSeq and Ensembl, and you have no evidence to suggest adding new isoforms. Even so, you should add the complete set of transcripts to the Editing Area to indicate that they have been reviewed. You can also provide additional information in the Annotation Information Panel, such as the gene symbol, description, and Gene Ontology. Although gene symbols are often provided with the RefSeq or Ensembl genes, you should check for the use of standard nomenclature established by the Vertebrate Gene Nomenclature Committee (Tweedie et al. 2021).

## Conclusion

AgAnimalGenomes.org is a genome browser resource for viewing genes, variants, QTL, tissue-specific expression, and functional sequence annotation data generated by the FAANG Consortium for livestock species. The browsers, based on JBrowse and Apollo, support the modification and creation of new gene models. In addition to the Apollo built-in features that aid manual annotation, we provide various track visualizations to help annotators discern gene structure alternatives. The Faceted Track Selector supports flexible searching to select tracks for viewing from among hundreds of experiments.

With the AgAnimalGenomes genome annotation tools, we hope to build a community of researchers who wish to contribute to the improvement of the gene catalogs of livestock species. The tools will also be useful to investigators wishing to verify and possibly correct genes that are important in their research. The Gene Prediction Problem tracks, showing thousands of genes per species that are discordant between Ensembl and RefSeq, will alert researchers to gene model issues that may affect data interpretation and help annotators focus on genes needing refinement. Furthermore, the annotation tutorial and tools at AgAnimalGenomes.org can serve as educational resources for students in genetics, genomics, and animal science. We have described the most common annotation scenarios to help users get started, but with experience, annotators will develop their own approaches to solve a wide variety of gene prediction issues. New annotators who want to test Apollo features should not be concerned about making mistakes, because annotations can be deleted and any changes can be reversed. We encourage the research community to use the annotation tools at AgAnimalGenomes.org and we welcome comments or suggestions for improvement.

## Methods

We created new genome browsers for the following genome assemblies: *Bubalus bubalis* NDDB_SH_1, *Capra hircus* ARS1, *Gallus gallus* GRCg6a, *Equus caballus* EquCab3.0, *Ovis aries* ARS-UI_Ramb_v2.0, and *Sus scrofa* Sscrofa11.1. The *Bos taurus* ARS-UCD1.2 genome browser was previously described (Triant et al. 2020), as part of the Bovine Genome Database website (Shamimuzzaman et al. 2020), and was updated for inclusion in AgAnimalGenomes. Genome assemblies and RefSeq gene sets were downloaded from NCBI (https://ftp.ncbi.nlm.nih.gov/genomes/refseq/vertebrate_mammalian/) (Sayers et al. 2021). Ensembl gene sets were downloaded from Ensembl (https://ftp.ensembl.org/pub/release-107/gff3/) (Cunningham et al. 2022). QTL data in gff format were downloaded from AnimalQTLdb (Release 47) (Hu et al. 2022). Variant data were downloaded from Ensembl Variation (https://ftp.ensembl.org/pub/release-107/variation/vcf/) (Cunningham et al. 2022) and the European Variation Archive (https://www.ebi.ac.uk/eva/?RS-Release&releaseVersion=4) (Cezard et al. 2022).

RNAseq data from Bioprojects listed in Table 2 were obtained from the NCBI Sequence Read Archive. Processing of some of the bovine RNAseq data was previously reported in (Triant et al. 2020), and similar methods were used to process the remainder of the data listed in Table 1. Briefly, reads were adapter and quality trimmed with Fastq-MCF (https://code.google.com/p/ea-utils/wiki/FastqMcf) and DynamicTrim (Cox et al. 2010), respectively, and aligned to unmasked genome assemblies with Hisat2 (Kim et al. 2015). StringTie was used to assemble RNAseq read alignments into transcripts, and output was converted to GFF3 format using gffread. RegTools (Cotto et al. 2023) was used to create bed files of RNAseq junctions. RNAseq visualization tracks were created using JBrowse utilities (Buels et al. 2016).

Datasets used in functional sequence annotation listed in Table 2 were downloaded from various sources. ChIP-seq peaks (BED format) for equine histone modification marks (Kingsley et al. 2019, 2021) were downloaded from the FAANG Data Portal (https://data.faang.org/dataset/PRJEB35307 and https://data.faang.org/dataset/PRJEB42315) (Harrison et al. 2021). Peak files in BED format for ATAC-seq, ChIP-seq, DNase Hypersensitivity, and chromatin states for bovine, chicken, and pig (Kern et al. 2021; Pan et al. 2021) were downloaded from a server at University of California-Davis (https://farm.cse.ucdavis.edu/~ckern/Nature_Communications_2020/ and https://farm.cse.ucdavis.edu/~zhypan/Nature_Communications_2021/). ATAC-seq peaks in BED format for goat and pig (Foissac et al. 2019) were downloaded from the Fr-AgEncode website (http://www.fragencode.org/results.html).

Metadata (tissue, Biosample id, SRA experiment accession, Bioproject accession) for the tissue-specific data were curated from NCBI Biosample, NCBI SRA, and EBI Biosamples (Courtot et al. 2022; Sayers et al. 2023a). We manually assigned Brenda Tissue Otology (BTO) (Chang et al. 2021) terms to samples. UBERON terms (Haendel et al. 2014) for samples were either obtained from EBI Biosamples or manually assigned. We also manually assigned organ system(s) to each sample based on UBERON. To facilitate the easy viewing of tracks representing different experiments for the same tissue sample, we created an identifier called *Specimen Tag* for tissue-specific tracks. In some cases, the Specimen Tag is identical to the Biosample id. For cases in which different libraries of the same individual sample were submitted to NCBI under different Biosample accessions, we created the Specimen Tag by combining the tissue name with the sample individual or replicate id.

Genome browsers were set up using Apollo 2.7.0 (Dunn et al. 2019), which is a plugin for JBrowse 1 (Buels et al. 2016). BLAST was set up using SequenceServer (Priyam et al. 2019) and configured to enable viewing alignments to genomes in JBrowse and Apollo.

## How to cite

All of the track data provided in our browsers have been previously reported (Tables 1 and 2). If you use an AgAnimalGenomes browser in an analysis for publication, you should cite not only this paper, but the relevant genome assembly paper as well as tracks used in the analysis. To help users credit the data sources, a table provided on the *How To Cite* page provides references and links to PubMed. For tissue-specific tracks, such as RNAseq and functional annotation data, the Bioproject provided in the Faceted Track Selector can be used to look up the publication on the *How To Cite* page.

## Supplementary Information

Below is the link to the electronic supplementary material.

Supplementary file1 (PDF 7987 KB)

## Data Availability

The tools described in this paper are freely accessible at http://aganimalgenomes.org/.
