# Supplementary material for: AgAnimalGenomes: browsers for viewing and manually annotating farm animal genomes
Source: Mamm Genome. 2023 Jul 17;34(3):418–36. doi: 10.1007/s00335-023-10008-1 (PMC10382368; doi:10.1007/s00335-023-10008-1)
Supplement: Supplementary file 1 — Supplementary file1 (PDF 7987 KB) [file 335_2023_10008_MOESM1_ESM.pdf]

AgAnimalGenomes: Browsers for Viewing and Manually Annotating Farm Animal Genomes

Mammalian Genome

Deborah A. Triant, Amy T. Walsh, Gabrielle A. Hartley, Bruna Petry, Morgan R. Stegemiller, Benjamin M. Nelson, Makenna M. McKendrick, Emily P. Fuller, Noelle E. Cockett, James E. Koltes, Stephanie D. McKay, Jonathan A. Green, Brenda M. Murdoch, Darren E. Hagen, Christine G. Elsik

Corresponding author:
Christine Elsik
Division of Animal Sciences, University of Missouri, Columbia, MO 65201, USA
elsikc@missouri.edu

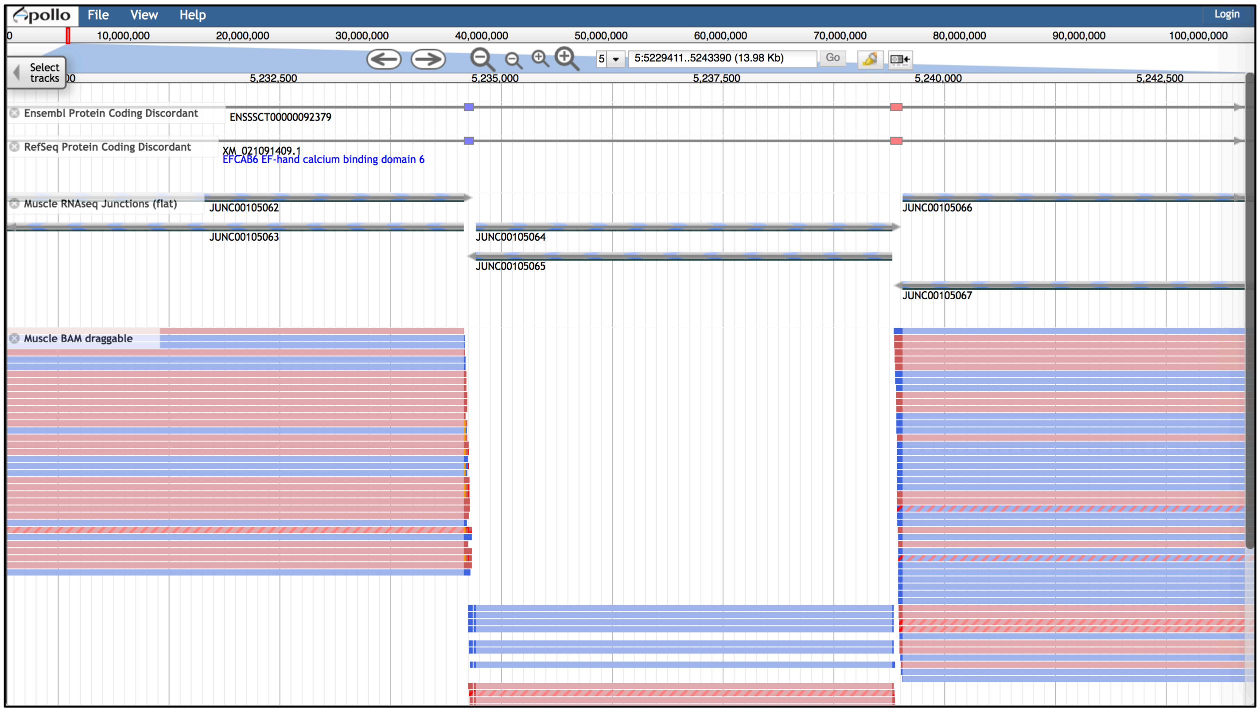


Fig. S1. RNAseq tracks shown as flat junctions and read alignments (BAM) in the draggable format. The zoom level must be sufficient to avoid the “Too much data to show” error. Blue and red colors in the BAM track represent the strand of the read alignment. Spans between aligned read segments are shown in lighter red and blue. The darker blue and red aligned portions of the reads are barely visible. The reads with hash marks are paired-end reads with a missing paired read.

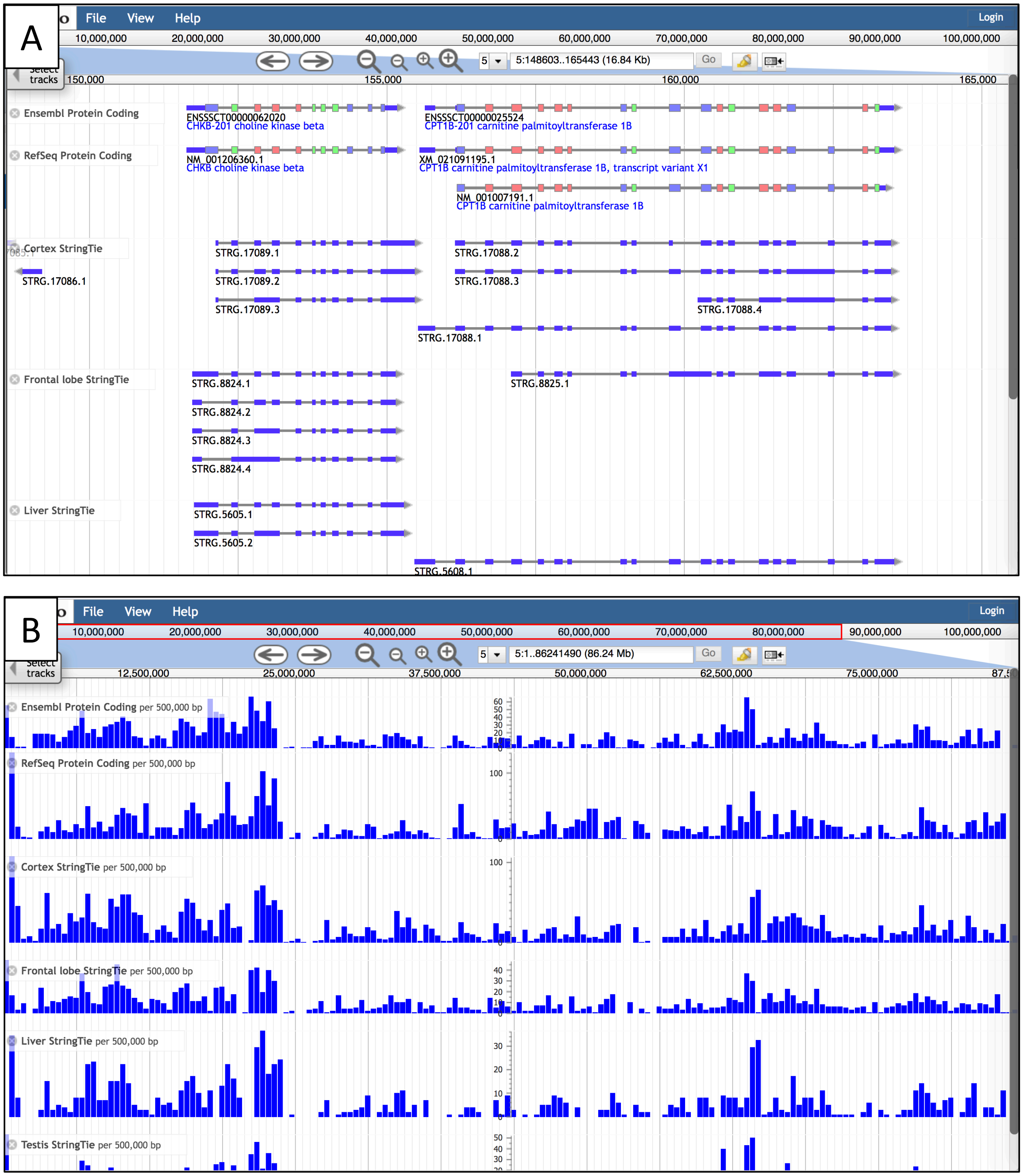


Fig. S2. A) StringTie tracks, zoomed-in. Notice all the exons in StringTie tracks are shown as dark blue (noncoding RNA) because the coding potential is not known. B) StringTie tracks zoomed out.

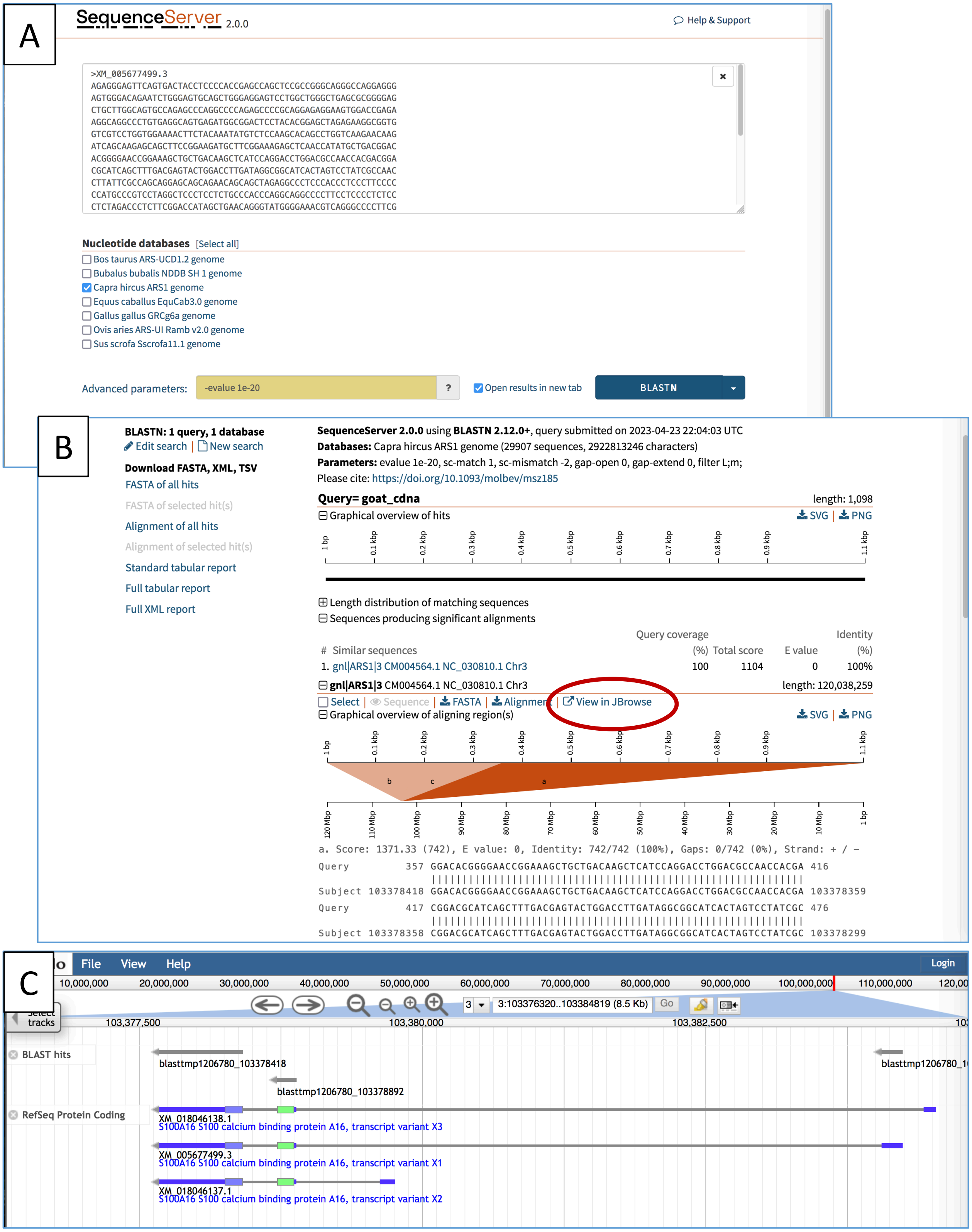


Fig. S3. A) The BLAST search interface. B) The results page. Clicking "View in JBrowse" (circled in red on the figure) opens JBrowse in a new browser tab. C) BLAST HSPs showing in JBrowse. If you are already logged into Apollo, the Apollo view, rather than JBrowse, will open.

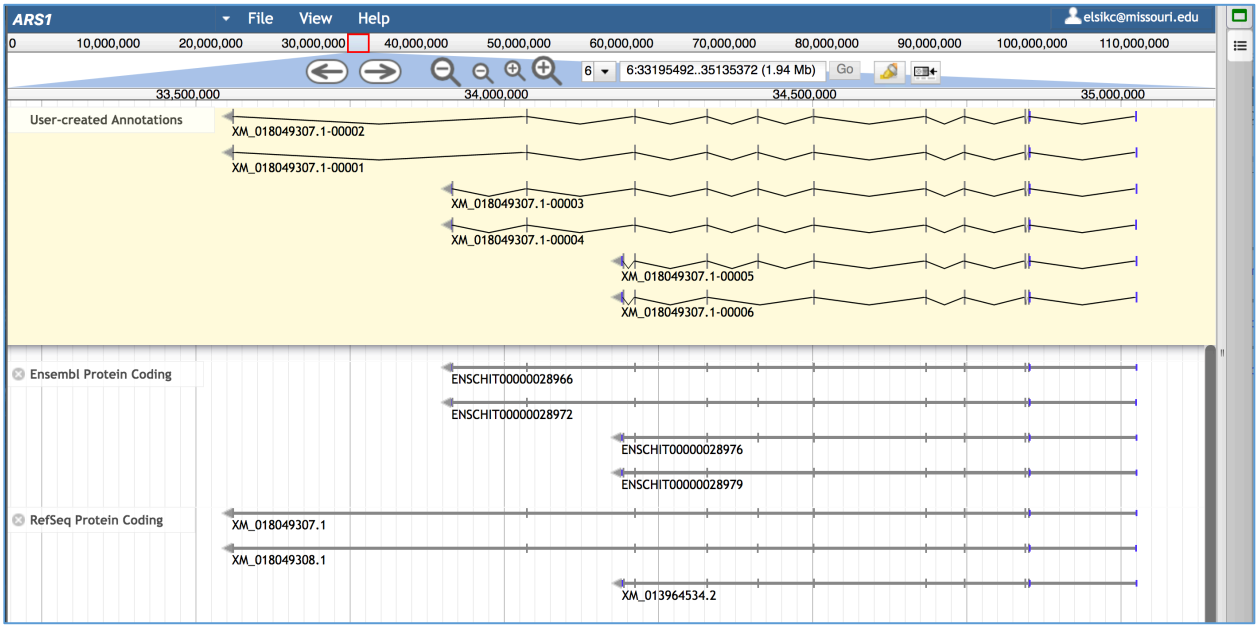


Fig. S4. Transcripts that have been dragged to the Editing Area. XM_018049307.1 was the first transcript to be added, so this identifier is used as the gene name in the Annotation Information Panel and the base name for all the transcripts, including those initiated with Ensembl transcripts.

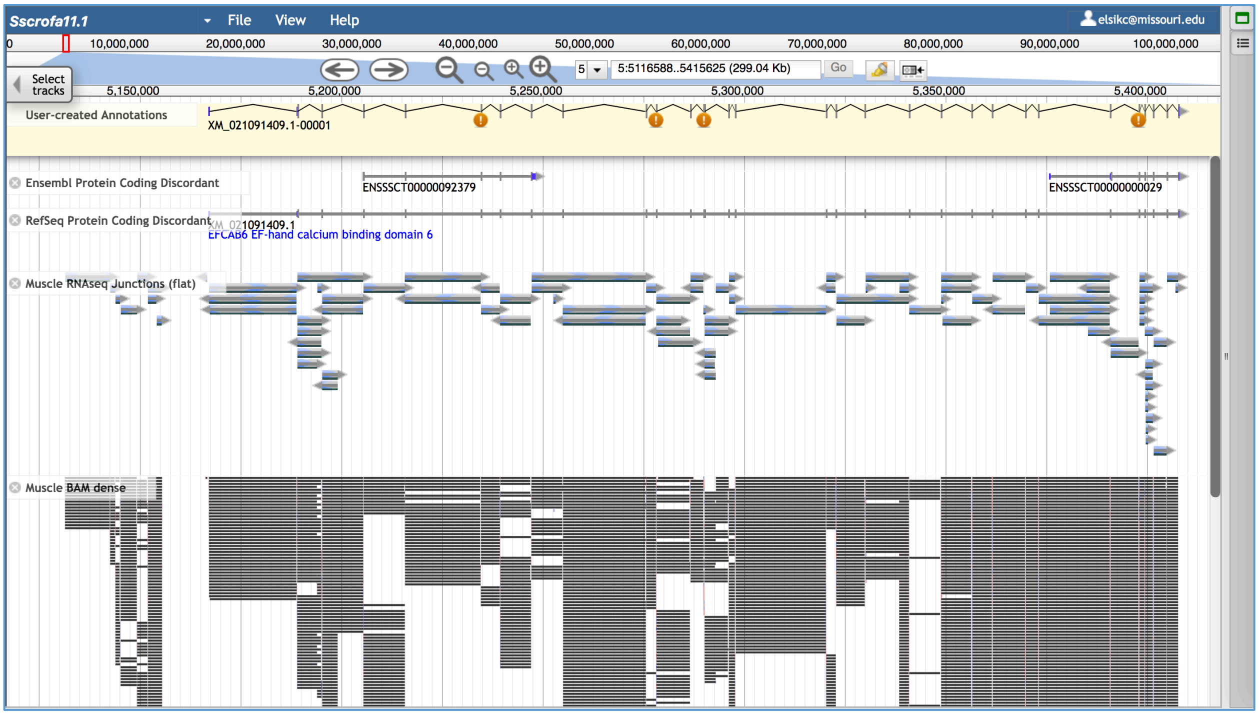


Fig. S5. An annotation with non-canonical splice sites highlighted by exclamation marks. This also shows the tracks that are usually used to verify splice sites (flat RNAseq junctions and dense RNAseq BAM read alignments).

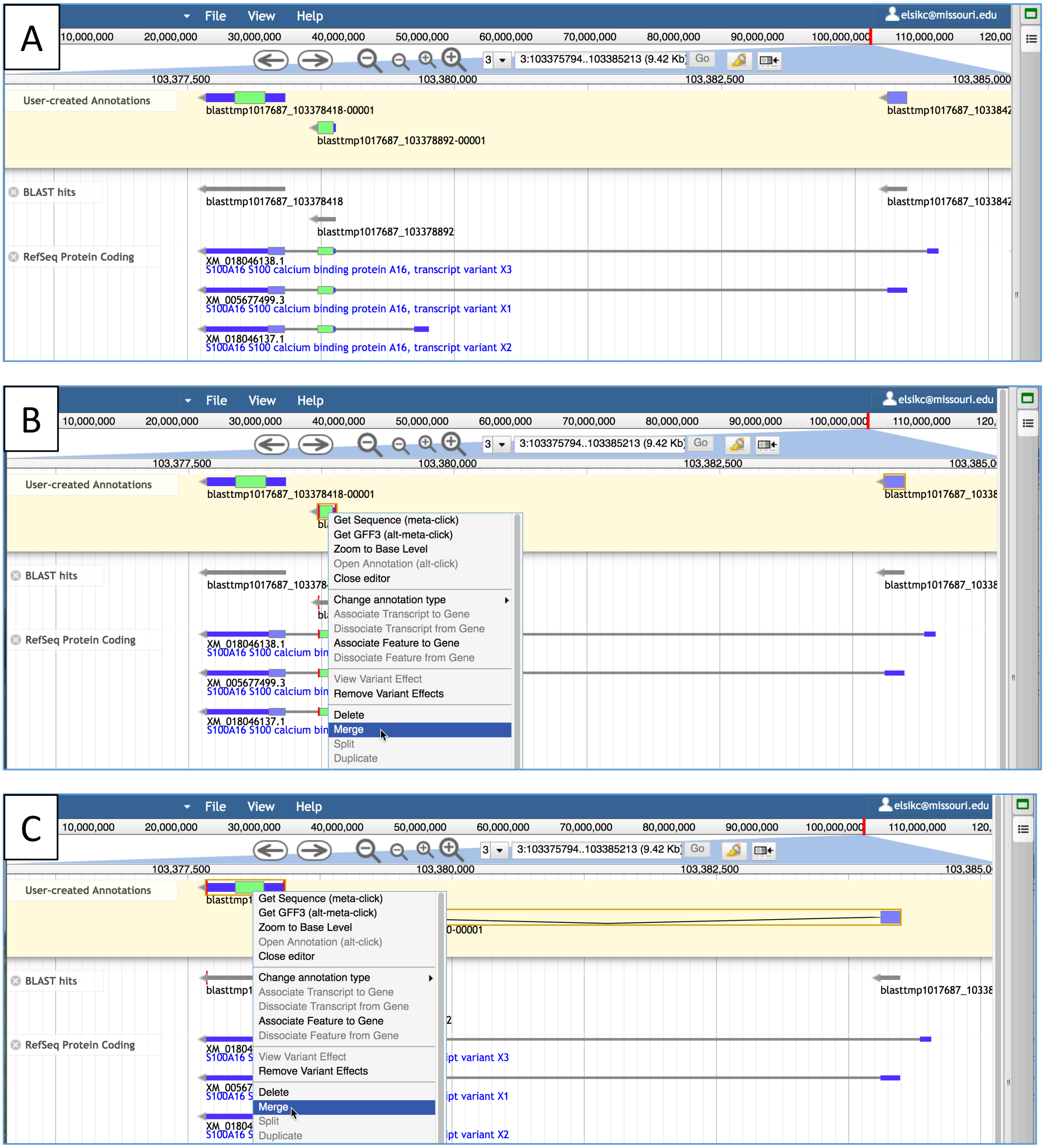


Fig. S6. A) BLAST HSPs have been dragged to the Editing Area to create an annotation, following from the BLAST search shown in Fig. S3. B) After clicking the 5’ exon (shown surrounded by a gold box on the far right), the middle exon is right-clicked and “Merge” is selected. C) After the two are merged in panel B, the same procedure is used to merge those to the 3’ exon (on the far left).

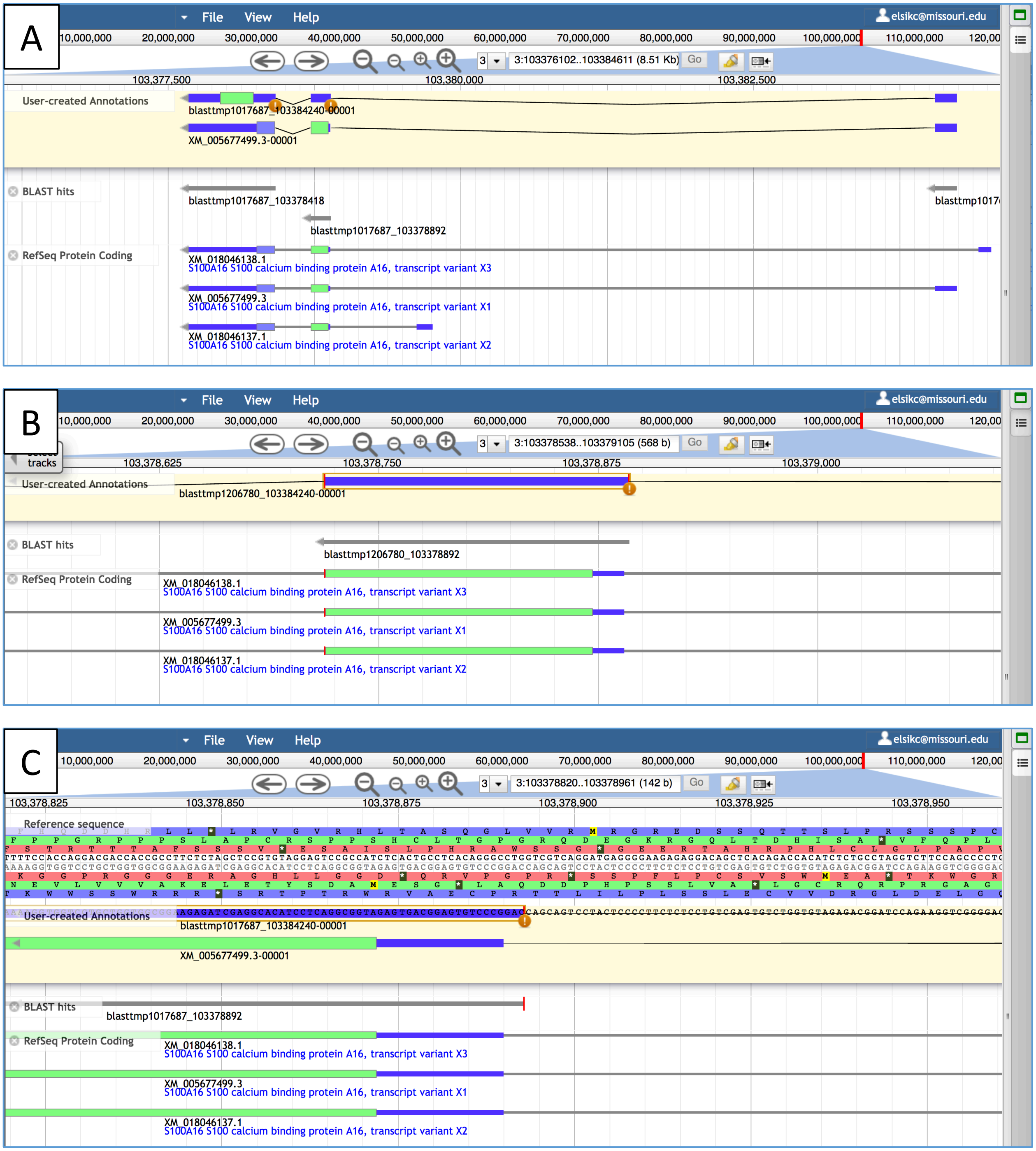


Fig. S7. The annotation resulting from merging BLAST HSPs. A) For this example, the BLAST search had been performed using the sequence of the NCBI transcript XM_005677499.3 in order to demonstrate some issues with BLAST HSPs, so we have added the NCBI annotation transcript to the Editing Area for comparison. The annotation has non-canonical splice sites and exon reading frames do not match those of the NCBI transcript. B) A zoomed-in view of middle exon, which has been clicked to highlight matching exon edges in other tracks. It matches the BLAST hit, but not the RefSeq transcripts. C) The exon edge has been clicked so that it can be dragged to the correct position.

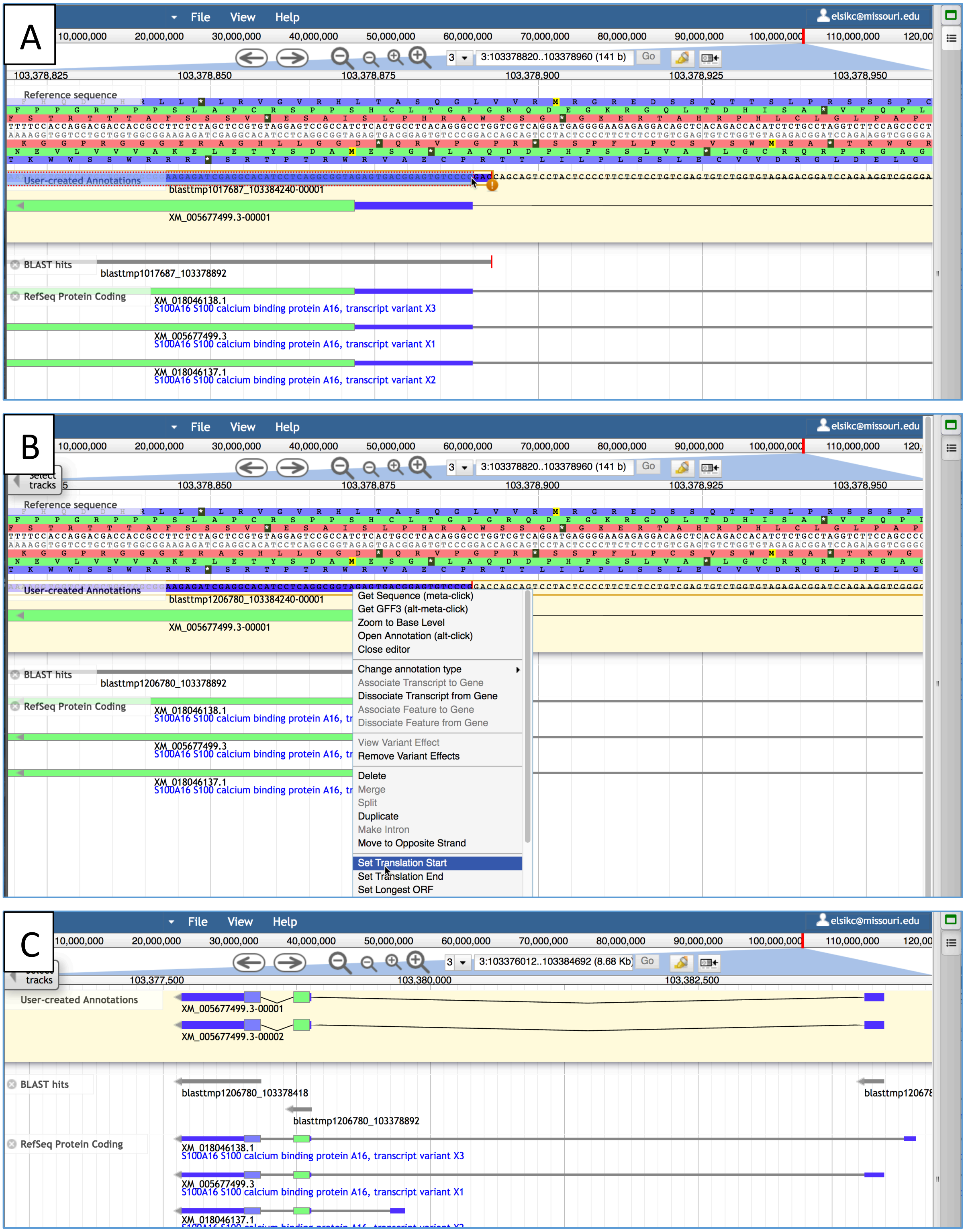


Fig. S8. Corrections to the annotation created by merging BLAST HSPs. A) The edge in the middle exon is corrected by dragging it to the left. B) The translation start is reset. The last correction, not shown, is a correction to the left edge of the 5’ exon. C) The corrected annotation. Once the corrections resulted in a CDS with a reading frame that matched the corresponding CDS in the NCBI transcript, the base name of the BLAST-based annotation was automatically changed to the NCBI id.
